# Impact of grafting using thin upper pole artery ligation on living-donor adult kidney transplantation: The STROBE study

**DOI:** 10.1097/MD.0000000000005188

**Published:** 2016-10-21

**Authors:** Takahisa Hiramitsu, Manabu Okada, Kenta Futamura, Takayuki Yamamoto, Makoto Tsujita, Norihiko Goto, Shunji Narumi, Yoshihiko Watarai

**Affiliations:** Department of Transplant and Endocrine Surgery, Nagoya Daini Red Cross Hospital, Myoken-cho, Showa-ku, Nagoya, Aichi, Japan.

**Keywords:** kidney graft function, living-donor kidney transplantation, thin upper pole artery ligation

## Abstract

This study aimed to investigate the impact of grafting using thin upper pole artery ligation for living-donor adult kidney transplantation. Few reports have examined the safety of thin upper pole artery ligation.

Between January 2008 and May 2015, 613 consecutive living-donor adult kidney transplantations were performed. We excluded 21 recipients who experienced graft loss due to factors that were unrelated to surgical complications and 3 recipients with grafts treated with arterial reconstruction and thin upper pole artery ligation for 3 arteries. We included 439 kidney grafts with single arteries (Single Artery Group), 123 with reconstructed arteries (Arterial Reconstruction Group) and 27 with ligated thin upper pole arteries (Arterial Ligation Group) in this retrospective cohort study. To evaluate the safety of thin upper pole artery ligation, we compared the Arterial Ligation Group with the Single Artery and Arterial Reconstruction groups. We evaluated the characteristics of the enrolled donors, recipients, and their grafts. Thereafter, we investigated recipients’ perioperative and postoperative estimated glomerular filtration rate (eGFR) and complication rates.

Significant differences among the 3 groups were identified for donor sex and endoscopic nephrectomy rates. Recipient eGFR and the complication rates were adjusted according to these factors. The perioperative and postoperative eGFR of recipients did not differ significantly in the Arterial Reconstruction and Single Artery groups with low complication rates.

Thin upper pole artery ligation is a safe procedure for living-donor adult kidney transplantation and may prevent unnecessary arterial reconstruction and associated complications.

## 1 Introduction

Reportedly, 18% to 30% of potential kidney donors require grafting of >2 arteries unilaterally and 15% require grafting of >2 arteries bilaterally.^[1]^ Grafting with >2 arteries is sometimes required after endoscopic donor nephrectomy.^[2]^ The safety and efficacy of arterial reconstruction has been the topic of several reports.^[3–6]^ Several reports have recommend the reconstruction of the lower pole arterial branches to prevent ureteral complications.^[5,7]^ On the other hand, there have not been any reports on the importance of preserving the thin upper pole arteries. Some grafts have very thin upper pole arteries that feed small areas and that are very thin to reconstruct. Most arterial reconstructions are performed using the conjoined, end-to-side, and interposition methods. For grafts of very thin upper pole arteries, arterial reconstruction presents a risk of arterial complications. To prevent arterial complications, thin upper pole artery ligations are performed instead. However, very few reports have discussed the safety of thin upper pole artery ligation.^[8]^ Thus, we investigated the safety of grafting using thin upper pole artery ligation in living-donor adult kidney transplantation.

## 2 Methods

### 2.1 Ethics review

This study was approved by the Institutional Review Board of Nagoya Daini Red Cross Hospital, Aichi, Japan, and was conducted according to the Declaration of Helsinki.

### 2.2 Study design

To investigate the safety of thin upper pole arterial ligation, adult recipients of living-donor kidney transplantation were divided into 3 groups: the Arterial Ligation, Arterial Reconstruction, and Single Artery groups. The perioperative and postoperative estimated glomerular filtration rate (eGFR) and complication rates in the Arterial Ligation Group were compared with those of the 2 other groups. This retrospective cohort study was conducted according to the Strengthening the Reporting of Observational Studies in Epidemiology guidelines.

### 2.3 Participants

**Figure 1:**
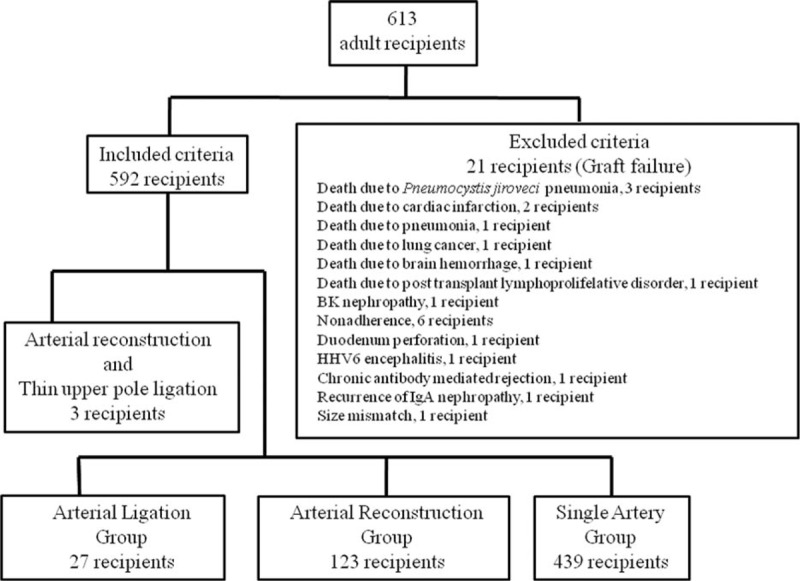
Patient flow chart.

Between January 2008 and May 2015, 613 consecutive living-donor adult kidney transplantations were performed at our hospital and included in this study. The recipients were observed every month between January 2008 and May 2015 (mean observation period: 43.3 ± 24.9 months). Twenty-one recipients dropped out of the study with graft failure unrelated to surgical complications (Fig. 1).

In total, 592 recipients were followed up. However, 439 of 592 kidney grafts were single artery (Single Artery Group), and 123 kidney grafts underwent arterial reconstruction (Arterial Reconstruction Group). Thin upper pole arteries (<2 mm) were ligated in 27 kidney grafts (Arterial Ligation Group). Three kidney grafts were excluded because they required arterial reconstruction and thin upper pole graft artery ligation for a total of 3 arteries (2 arteries and 1 thin upper pole artery) (Fig. 1). The outcomes were evaluated by examining the perioperative and postoperative eGFR and complication rates of the recipients. Arterial thrombosis, urine leakage, ureterial stricture, delayed graft function, postoperative bleeding, lymphocele, acute cellular rejection, and antibody-mediated rejection were investigated. Patient data were retrospectively collected from patients’ charts, and there were no missing data.

### 2.4 Statistical analysis

Statistical analyses were performed using the analysis of variance for continuous data and the chi-square exact test for categorical variables. Bonferroni correction was used for multiple comparison analyses. The generalized linear model analysis (gamma with log link) was used to make comparisons between surgical procedures and operative results. Generalized estimating equation (GEE) analysis was used to make comparisons between surgical procedures and longitudinal data on eGFR. The risk ratio was used to make comparisons between surgical procedures and incidence of complication rates. The analyses were performed using donor sex and laparoscopic donor nephrectomy as adjustment factors. *P* values of <0.05 were considered statistically significant. Analyses were performed using SAS package 9.0 (SAS Institute, Cary, NC).

### 2.5 Preoperative evaluation of graft arteries and indications for thin upper pole artery ligation

To select the reconstruction method, the number and size of graft arteries were preoperatively evaluated using 3-dimensional computed tomography (3D-CT). Ligation was indicated for very thin upper pole arteries that posed a risk of arterial complications (<2 mm). Lower pole graft arteries evident in 3D-CT images were always reconstructed to avoid ureteral complications.

### 2.6 Perfusion area of each ligated upper pole artery

The perfusion area of each ligated upper pole artery was estimated during cold perfusion. In all kidney grafts, the perfusion area of each ligated upper pole artery was <5%.

### 2.7 Arterial reconstruction methods

In general, 3 types of arterial construction methods were used for grafts with >2 arteries in the Arterial Reconstruction Group: the conjoined, end-to-side, and interposition methods.

### 2.8 Definition of time to initial urination

The time to initial urination was defined as the interval between blood reperfusion and the initial urination from the “graft” ureter.

## 3 Results

### 3.1 Participants

We observed 589 recipients every month between January 2008 and May 2015 (mean observation period: 43.3 ± 24.9 months) at our hospital; no patients dropped out during this period. Four hundred thirty-nine of the 589 kidney grafts had a single artery, and arterial reconstructions were performed in 123 kidney grafts. Thin upper pole artery ligation was performed in 27 kidney grafts.

### 3.2 Excluded recipients

A total of 24 recipients were excluded from this study, 21 due to graft failure and 3 due to a combination of arterial reconstruction and thin upper pole artery ligation. Among the 21 recipients, no thin upper pole artery ligation was identified. One graft underwent arterial reconstruction for 2 arteries, and 20 grafts were with a single artery. One recipient with arterial reconstruction dropped out from following death from pneumonia.

### 3.3 Descriptive data

We compared the characteristics of recipients and donors among the Arterial Ligation Group, the Arterial Reconstruction Group, and Single Artery Group (Table 1). We did not identify any significant differences in the characteristics of the recipients. The donor sex and endoscopic donor nephrectomy rates significantly differed among the groups. The body mass index (BMI) of the recipients and donors was similar among the groups. The mean eGFR of donors was also similar. The multiple comparison analysis in Table 2 shows a significant difference in donor sex and endoscopic donor nephrectomy rates between the Arterial Ligation Group and the Single Artery Group. All patient data collected were complete and accurate.

**Table 1:**
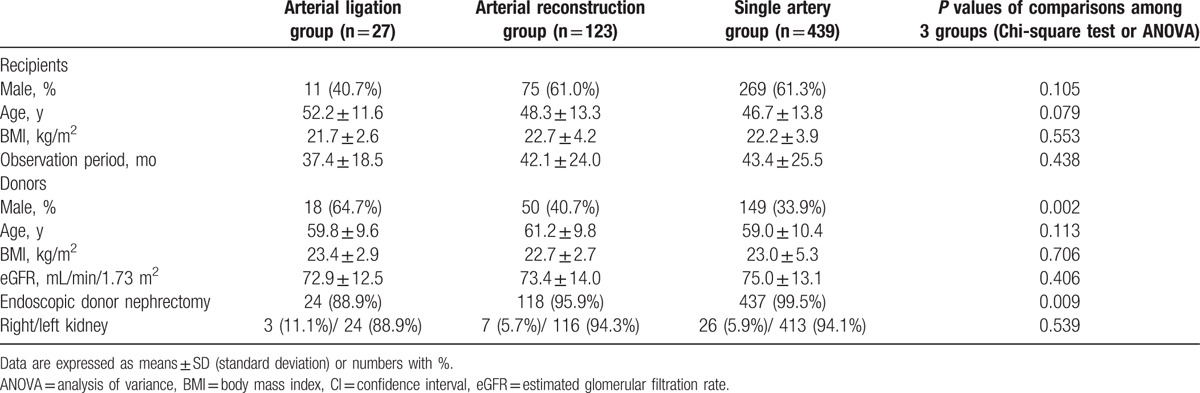
Characteristics of recipients and donors.

**Table 2:**
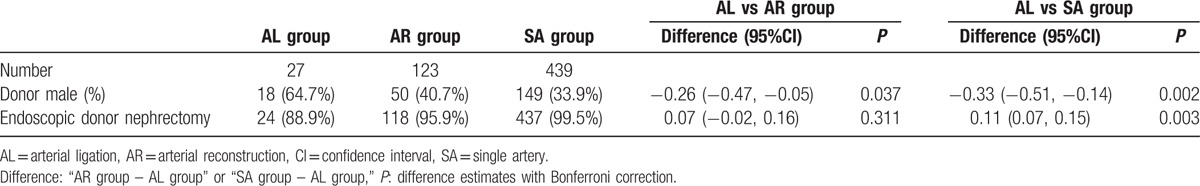
Comparison of characteristics.

### 3.4 Characteristics of kidney grafts and results of surgeries

The attributes of all kidney grafts are shown in Table 3. Kidney weights were similar among the groups. In the 3D-CT images, the mean diameter of the ligated arteries in the Arterial Ligation Group was 1.82 ± 0.36 mm, which was significantly thinner than that of the reconstructed arterial branches in the Arterial Reconstruction Group.

**Table 3:**
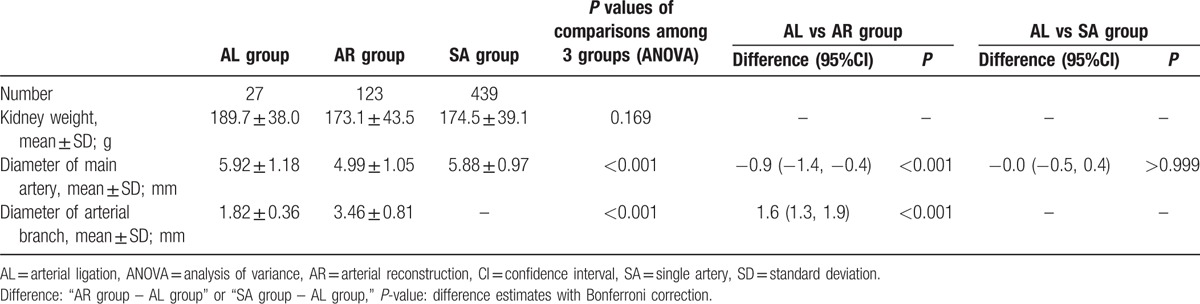
Characteristics of the kidney grafts.

Operative results are shown in Table 4. Donor sex and endoscopic donor nephrectomy rates were significantly different according to the characteristics of recipients and donors. Operative results were adjusted with these factors in mind. There were no differences in the operative duration, blood loss of donor nephrectomy, and initial urination among the groups. The warm ischemia time (WIT; interval between arterial clamping and the beginning of cold perfusion) and total ischemia time (TIT; interval between arterial clamping and blood reperfusion) were significantly longer in the Arterial Reconstruction Group.

**Table 4:**
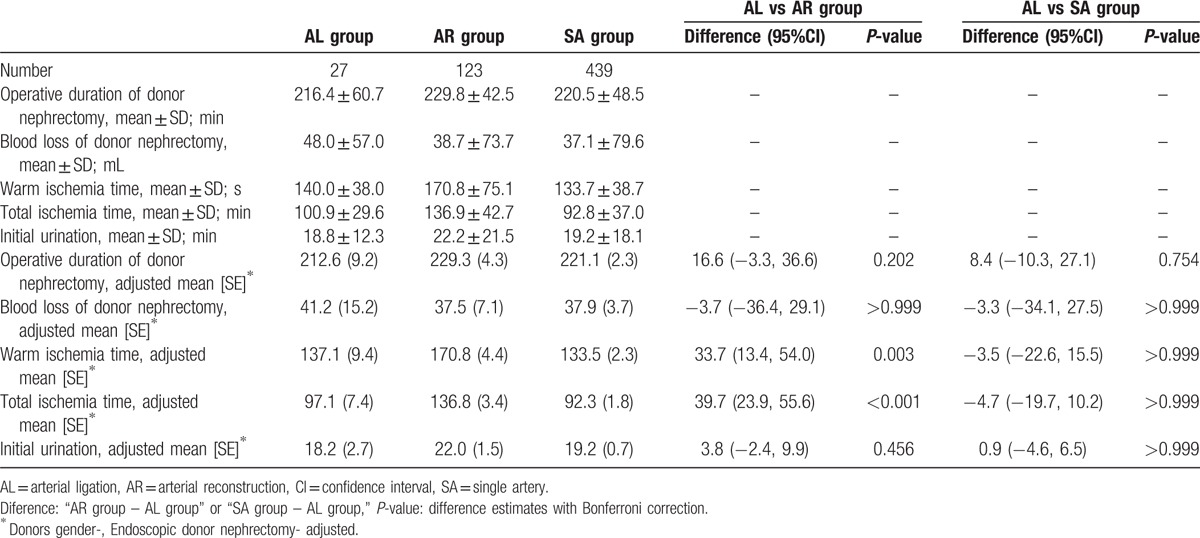
The results of the operation.

Complications that occurred in each group are shown in Table 5. Incidences of each complication (adjusted for donor sex and endoscopic operation rates) were not significantly different among the Arterial Ligation Group and the other groups.

**Table 5:**
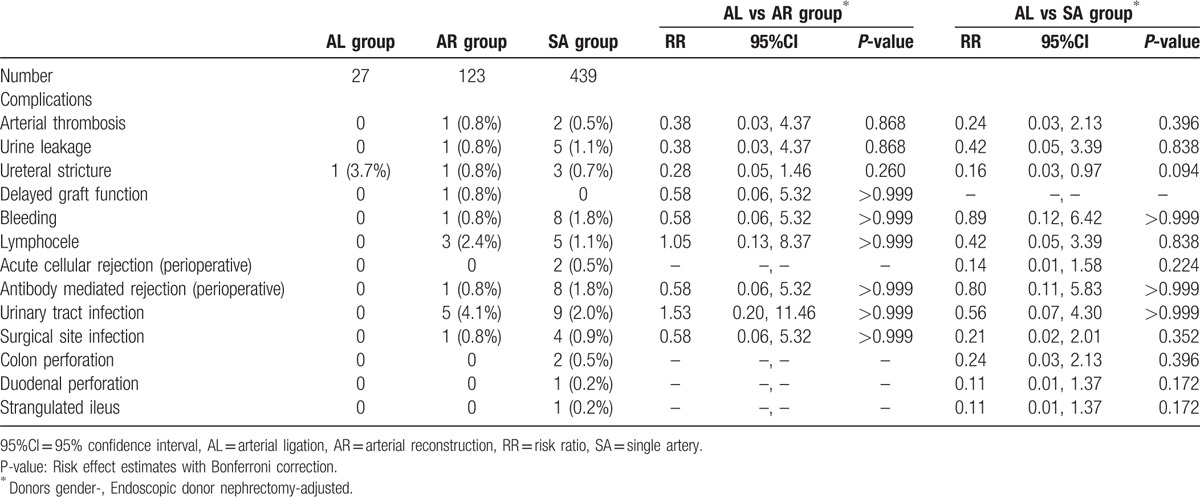
Complications in each group.

**Figure 2:**
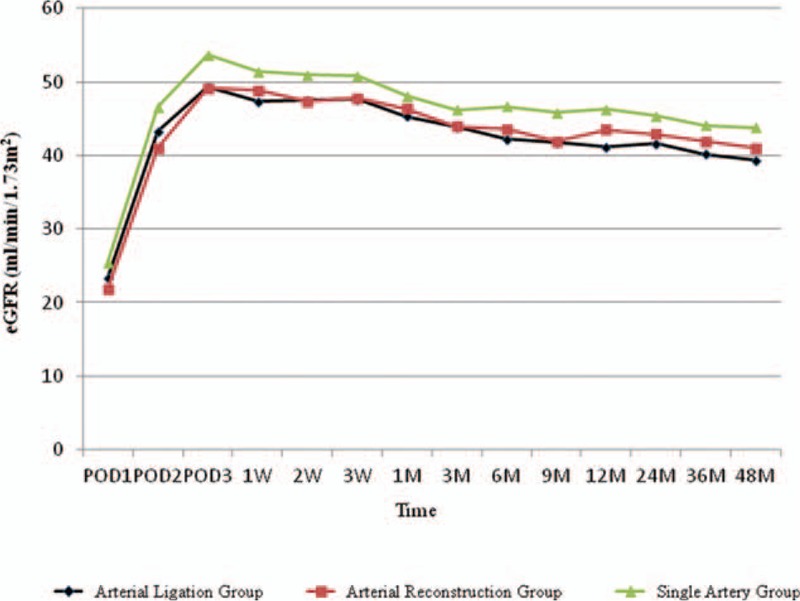
Mean estimated glomerular filtration rate of the recipients during the observation period (unadjusted).

**Figure 3:**
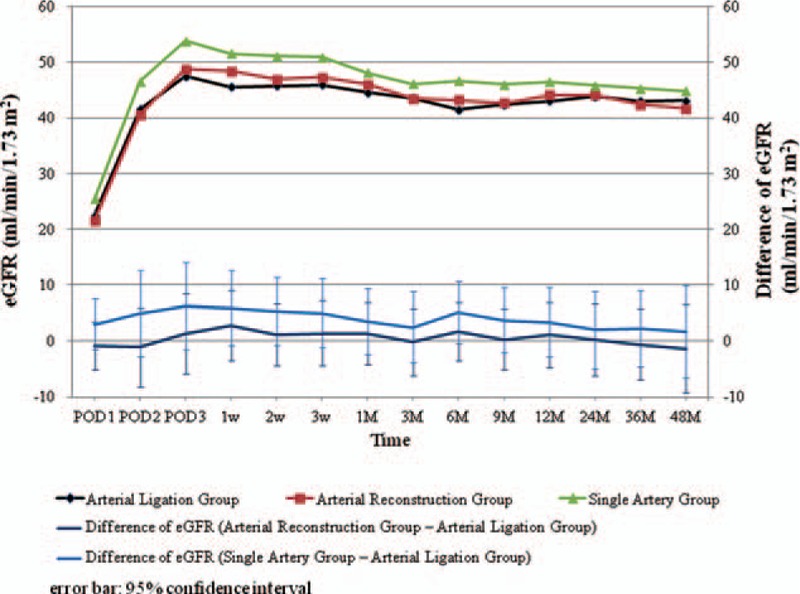
Mean and differences in the estimated glomerular filtration rate of the recipients during the observation period. There was no significant difference among the Arterial Ligation Group and the Single Artery and Arterial Reconstruction groups. eGFR = estimated glomerular filtration rate, POD = postoperative day.

The unadjusted eGFR of 3 groups are shown in Fig. 2. The eGFR and eGFR differences adjusted for donor sex and endoscopic operation rates are shown in Fig. 3. Differences in eGFR were not significantly different among the Arterial Ligation Group and the other groups throughout the entire observation period (Table 6).

**Table 6:**
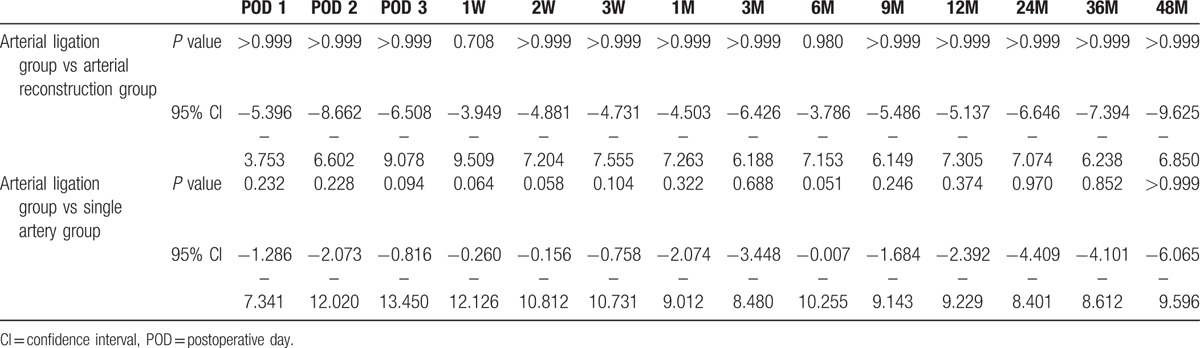
Perioperative and postoperative graft function.

## 4 Discussion

Using 3D-CT, it is easy to evaluate the number and size of kidney graft arteries preoperatively.^[9–15]^ Studies have reported that the preservation of the arterial branches that feed the lower pole of the kidney graft is important to prevent ureteral complications such as ureteral leakage and stricture.^[7]^ The safety of arterial branch reconstruction has been established.^[3–6]^ However, very thin upper pole arteries are sometimes present,^[15]^ and these are often ligated to shorten the TIT and prevent complications related to reconstruction. However, the safety of ligating these thin upper pole arteries is unclear. In this study, the mean diameter of 27 thin upper pole arteries was 1.82 mm, which was significantly thinner than that of the reconstructed arterial branches. We identified significant differences according to the donor sex and endoscopic nephrectomy rates. For accurate analysis, operative results, recipients’ eGFRs, and complication rates were adjusted according to these factors. No significant differences in operative results were demonstrated, except for WIT and TIT. The significantly longer WIT and TIT evident in the Arterial Reconstruction Group were reasonable because dealing with >2 arteries during donor nephrectomy and their reconstruction was more time consuming. Although the WIT was significantly longer in the Arterial Reconstruction Group, the mean WIT was 140 s in the Arterial Ligation Group and 171.9 s in the Arterial Reconstruction Group. This difference in WIT of only 31.9 s may have been too small to yield specific complications.^[16]^ Although the ligation of thin upper pole arteries significantly shortened the mean TIT in the Arterial Ligation Group compared to that in the Arterial Reconstruction Group, the perioperative eGFR, the time to initial urination, the incidence of delayed graft function, and the incidence of acute cellular rejection were similar between the Arterial Ligation and Arterial Reconstruction groups. The mean TIT was 100.9 min in the Arterial Ligation Group and 136.8 min in the Arterial Reconstruction Group. This difference was statistically significant, but the difference in TIT of only 35.9 min may have been too small to constitute a clinical difference.^[17]^ With regard to other adjusted complications, arterial thrombosis did not occur in the Arterial Ligation Group, and occurred in only 1 recipient in the Arterial Reconstruction Group. This implies that the ligation of thin upper pole arteries minimized the risk of arterial thrombosis in the Arterial Reconstruction Group. The mean diameter of the 27 thin upper pole arteries examined in this study was only 1.82 mm, which may have been a risk factor for complications related to reconstruction. Although the safety of arterial reconstruction was established, further assessments of the criteria that indicate arteries for reconstruction and optimum techniques for this procedure are required.^[3]^ Incidences of other complications in the Arterial Ligation Group compared with those in the Arterial Reconstruction Group were not significantly different. Furthermore complication rates were similar for the Arterial Ligation Group and Single Artery Group. These results demonstrated low complication rates in the Arterial Ligation Group.

Infarction of a small area of the upper pole (<5%) can occur during ligation of thin upper pole arteries. Although this might influence the postoperative kidney function of recipients, we did not observe significant differences in eGFRs in the Arterial Ligation Group and the other groups in this study. This suggests that the areas fed by thin upper pole arteries were too small to have an impact on graft function. These facts demonstrate the safety of the ligation of thin upper pole arteries.

One limitation of this study is its retrospective nature; prospective randomized studies on the impact of the ligation of thin upper pole kidney graft arteries are required in the future.

In conclusion, the ligation of thin upper pole arteries (<2 mm) is a safe procedure with a low incidence of complications when performed on selected thin upper pole arteries.
